# Cisplatin inhibits the proliferation of Saos-2 osteosarcoma cells via the miR-376c/*TGFA* pathway

**DOI:** 10.17305/bjbms.2020.4485

**Published:** 2021-04

**Authors:** Yuan Wang, Yichao Wu, Awei Cai, Chengxiao Ma, Shang Cai, Hao Wang, Yukang Que, Shenglin Xu, Tangbing Xu, Yong Hu

**Affiliations:** 1 Department of Orthopedic Disease and Oncology Surgery, First Affiliated Hospital of Anhui Medical University, Hefei, China; 2 Department of Orthopedics, Fourth Affiliated Hospital of Anhui Medical University, Hefei, China

**Keywords:** Cisplatin, miR-376c, transforming growth factor alpha, TGFA, Saos-2, cell proliferation, cell growth, osteosarcoma

## Abstract

The transforming growth factor alpha (*TGFA*) gene is involved in the proliferation and metastasis of various tumors, but its role in cell sensitivity to cisplatin chemotherapy is unclear. In this study, we investigated the mechanism underlying inhibitory effects of cisplatin on growth and proliferation of osteosarcoma cells. Osteosarcoma and normal skeletal muscle tissues were collected from 26 patients by biopsy. *TGFA* was silenced or overexpressed in Saos-2 osteosarcoma cells by transfection with *TGFA*-shRNA or *TGFA* ORF clone, respectively. MiR-376c was inhibited or overexpressed by transfection of Saos-2 cells with miR-376c sponge or miR-376c mimics, respectively. Cell growth was analyzed by MTT assay and cell proliferation by BrdU assay. MiR-376c and *TGFA* mRNA expression was detected by quantitative reverse transcription PCR and *TGFA* protein expression by Western blot. The target relationship between miR-376c and *TGFA* was assessed by luciferase reporter assay. Both in osteosarcoma tissues and Saos-2 cells, miR-376c expression was significantly decreased and *TGFA* mRNA expression was significantly increased compared with control. Transfection of Saos-2 cells with *TGFA*-shRNA silenced *TGFA* expression and significantly inhibited cell growth and proliferation. *TGFA* mRNA and protein expression in Saos-2 cells significantly decreased with increasing cisplatin concentrations (2.5–10 mg/L). Transfection with *TGFA* ORF clone reversed the inhibitory effects of cisplatin on Saos-2 cell proliferation. Compared with cisplatin (10 mg/L) treatment alone, the combined treatment with cisplatin and miR-376c mimics inhibited the proliferation of Saos-2 cells more significantly. MiR-376c suppressed *TGFA* expression by directly interacting with its 3’ UTR region. Overall, cisplatin inhibited the proliferation of Saos-2 cells by upregulating miR-376c and downregulating *TGFA* expression.

## INTRODUCTION

Human health is seriously threatened by cancer. The World Health Organization reported 18.1 million new cancer cases and 9.6 million cancer-related deaths in 2018 [1]. In the past few decades, patients with osteosarcoma have been treated with various chemotherapeutic drugs in combination with surgical resection, which evidently increased their 5-year survival rate. Due to the development of neoadjuvant chemotherapy and pulmonary metastasis resection, patients with osteosarcoma are no longer subjected to amputation but to limb salvage surgery instead, and their tumor-free survival rate has increased to about 60%–70%. However, high-dose chemotherapeutic agents have toxic side effects, accompanied by tumor cell mutation, primary or secondary drug resistance, early pulmonary metastasis, or local recurrence [2]. Moreover, it is rather difficult or even impossible to perform surgery for patients with osteosarcoma in the spine, the pelvis, or other special body parts as well as for patients at the advanced stage with recurrence or multiple metastatic foci. These patients can only take high-dose chemotherapeutic drugs, but with unsatisfactory treatment outcomes. Therefore, much research has endeavored to develop effective therapies with mild side effects, among which targeted therapy based on molecular biology and genetics has attracted widespread attention.

Cisplatin was first prepared in 1844, and in 1967 it was found to exert strong inhibitory effects on the growth of tumor cells [3]. Currently, cisplatin is used in 70%–80% of chemotherapeutic or combined methods for malignant tumors. However, the development and application of platinum drugs are limited because of structural similarity that leads to resistance or cross-resistance. Therefore, it is necessary to clarify the mechanism by which cisplatin works and to augment the sensitivity of osteosarcoma towards chemotherapy by developing targeted drugs.

Peptide growth factors are potent cell growth regulators. By forming complex biological networks peptide growth factors help exchange information between cells, and they can stimulate cell growth through autocrine, inhibit cell ­proliferation through paracrine, or promote cell differentiation and malignant transformation through endocrine signaling [4].

The *TGFA* gene, encoding transforming growth factor alpha (TGF-α), spans 70–100 kb, has 6 exons, and is located on the human chromosome 2. *TGFA* is upregulated in some human cancer cells, which is related to their metastasis and invasion. For example, *TGFA* can regulate the transformation of mammary epithelial cells, and TGF-α and epidermal growth factor receptor (EGFR) are overexpressed in human breast cancer tissues and various breast cancer cell lines [5]. EGFR antibody or specific kinase inhibitors can block the TGF-α/EGFR signaling pathway and prevent breast cancer growth induced by *TGFA* overexpression, suggesting that this pathway may play an important role in cancer treatment [6]. Wang et al. found that TGF-α regulated the proliferation and migration of KGN cells (derived from an invasive ovarian granulosa cell tumor [GCT]) via multiple signaling pathways, suggesting a key role of TGF-α in GCT growth and metastasis [7]. In addition, TGF-α affected the proliferation and metastasis of human osteosarcoma [1,8], having an oncogenic role in osteosarcoma growth [9].

MicroRNAs (miRNAs) are highly conserved endogenous, noncoding RNAs found in almost all organisms. They participate in many biological processes, including cell growth, development, apoptosis, proliferation, and differentiation. In animals, over 50% of miRNA genes in the genome are located in tumor-related or fragile sites [10].

MiRNAs inhibit or promote cell proliferation, tumor growth and metastasis by regulating oncogenes and tumor suppressor genes [11]. Members of the miR-376 family are abnormally expressed in many cancer types. As a member of this family, miR-376c has low expression in several types of cancer cells, and overexpression of miR-376c can inhibit cancer cell proliferation and metastasis. Zehavi et al. reported that miR-376c was obviously silenced in melanoma cells, and the overexpression of miR-376b and miR-376c inhibited insulin-like growth factor 1 receptor expression and suppressed cell proliferation and metastasis [12]. MiR-376c also showed low expression in osteosarcoma cells [13], indicating it may be involved in osteosarcoma cell proliferation and metastasis.

Although the *TGFA* gene is demonstrated to be involved in cancer cell proliferation and metastasis, its role in cell sensitivity to cisplatin chemotherapy remains unclear. MiR-376c has been shown to regulate the expression of *TGFA*, therefore, the sensitivity of cancer cells to cisplatin chemotherapy may be associated with the miR-376c/*TGFA* pathway. In this study, we investigated the mechanism underlying inhibitory effects of cisplatin on growth and proliferation of osteosarcoma cells, by analyzing the relationship between cisplatin, *TGFA*, and miR-376c. Our findings provide a valuable evidence for developing targeted drugs that would enhance the sensitivity of osteosarcoma to chemotherapeutic agents.

## MATERIALS AND METHODS

### Clinical samples

Fresh osteosarcoma tissues and normal skeletal muscle tissues were collected from patients receiving a biopsy in our hospital, immediately placed in liquid nitrogen, and stored at -80°C prior to use. Osteosarcoma was diagnosed by postoperative histopathological examination. The osteosarcoma tissues were collected from 20 males and 6 females. No patient received radiotherapy or chemotherapy before surgery. The collection process has been approved by the ethics committee of our hospital, and written informed consent has been obtained from all patients.

### Cells and reagents

Human osteosarcoma cell line Saos-2 and normal human osteoblasts hFOB were purchased from the Cell Bank of Shanghai Institutes for Biological Sciences, Chinese Academy of Sciences (China). *TGFA*-short hairpin (sh)RNA, *TGFA* open reading frame (ORF) clone, miR-376c sponge and miR-376c mimics plasmids were obtained from Wuhan Viraltherapy Technologies Co., Ltd. (China). Cisplatin was obtained from Sigma-Aldrich (USA).

The cells were cultured in Dulbecco’s Modified Eagle Medium [DMEM] (Sigma-Aldrich, USA) containing 10% fetal bovine serum [FBS] (Gibco, USA), 100 U/mL penicillin (Sigma-Aldrich, USA) and 100 U/mL streptomycin (Sigma-Aldrich, USA), and incubated in a 5% CO_2_ incubator at 37°C with a relative humidity of 95%. The medium was refreshed every 2–3 days until the cells adhered to the wall.

### Downregulation of TGFA expression by transfection with shRNA

Saos-2 cells transfected with empty vector were set as a blank group (control), those transfected with antisense sequence shRNA were set as a negative control group (control-shRNA), and those transfected with *TGFA* shRNA plasmid were set as an experimental group (*TGFA*-shRNA). Cells in the logarithmic growth phase were digested, counted, seeded in a 6-well plate at a density of 2.5 × 10^5^/well and placed in a 5% CO_2_ incubator at 37°C with saturated humidity. When the confluence reached 80%–90%, the cells were transfected with control-shRNA or *TGFA*-shRNA plasmids according to the manufacturer’s instructions for Lipofectamine^®^ 2000 reagent (Thermo Fisher Scientific, USA). Specifically, cell culture medium was replaced with serum-free medium 2 h before transfection. *TGFA*-shRNA plasmid [4 mg] (4 mg pSliencer™ purchased from Thermo Fisher Scientific [USA] for ­control-shRNA, and sterile water with the same volume for control) was diluted by 0.25 mL of serum-free high-glucose DMEM (Sigma-Aldrich, USA). Meanwhile, 10 μL of Lipofectamine^®^ 2000 reagent was diluted by 0.75 mL of high-glucose DMEM. The two dilutions were then fully mixed and incubated at room temperature for 20 min. The transfected cells were cultured for 4 h, and then the medium was replaced with complete medium. Saos-2 cells with silenced *TGFA* gene were finally obtained after G418 screening and passage.

The detailed procedure for G418 screening is shown below. After transfection, cells were cultured for 24 h, passaged in a 1:10 proportion, and further cultured. After the cells adhered to the wall, the culture medium was discarded, followed by washing with phosphate-buffered saline (PBS) and the addition of fresh G418 screening culture medium. According to the color of culture medium and cell growth state, the medium was refreshed every 3–5 days. In the case of considerable cell death, the concentration of G418 medium was halved to continue screening. After 10–14 days of screening, resistant clones appeared and drug treatment was stopped to allow the clones to further grow. The cell suspension was then prepared, and the cells were counted and diluted (1/10 μL) with culture medium. Subsequently, culture medium (150 μL/well) and the cell suspension (10 μL/well) were added into 96-well plates sequentially. Then the cells were transferred into 48-well plates and further cultured, from which total RNA was extracted for quantitative PCR.

### Construction of cells with a stable overexpression of TGFA

Saos-2 cells transfected with empty vector were set as a blank group (control), those transfected with pcDNA3.1 were set as a negative control group (pcDNA3.1), and those transfected with *TGFA* ORF clone plasmid were set as an experimental group (*TGFA*-ORF clone). When the confluence reached 80%–90%, the cells were transfected with pcDNA3.1 (Thermo Fisher Scientific, USA) or *TGFA* ORF clone plasmids according to the manufacturer’s instructions for Lipofectamine^®^ 2000 reagent. Specifically, cell culture medium was replaced with serum-free medium 2 h before transfection. *TGFA* ORF clone plasmid [4 μg] (4 μg pcDNA3.1 plasmid, and sterile water with the same volume for control) was diluted by 0.25 mL of serum-free high-glucose DMEM. Meanwhile, 10 μL of Lipofectamine^®^ 2000 reagent was diluted by 0.75 mL of high-glucose DMEM. The two dilutions were thereafter completely mixed and incubated at room temperature for 20 min. The transfected cells were cultured for 4 h, and then the medium was replaced with complete medium. Saos-2 cells with overexpressed *TGFA* gene were finally obtained after G418 screening and passage.

### Downregulation of miR-376c expression by transfection with miR-376c sponge

Saos-2 cells transfected with empty vector were set as a blank group (control), those transfected with antisense sequence vector were set as a negative control group (vector), and those transfected with miR-376c sponge plasmid were set as an experimental group (miR-376c sponge). When the confluence reached 80%–90%, the cells were transfected with vector or miR-376c sponge plasmids according to the manufacturer’s instructions for Lipofectamine^®^ 2000 reagent. Specifically, cell culture medium was replaced with serum-free medium 2 h before transfection. MiR-376c sponge plasmid [20 μg] (15 μg psPAX2 obtained from Sigma-Aldrich [USA] for vector, and sterile water with the same volume for control) was added together with 10 μg pMD2G (Sigma-Aldrich, USA) and Opti-MEM (Gibco, USA) into a final volume of 2.5 mL, and then incubated at room temperature for 5 min. Afterwards, 100 μL of Lipofectamine^®^ 2000 reagent was mixed with 2.4 mL of Opti-MEM and incubated at room temperature for 5 min. Subsequently, the two mixtures were mixed and incubated at room temperature for 20 min. The transfected cells were cultured for 4 h and then the medium was replaced with complete medium. Saos-2 cells with downregulated miR-376c expression were finally obtained after G418 screening and passage.

### Construction of cells with a stable overexpression of miR-376c

Saos-2 cells transfected with empty vector were set as a blank group (control), those transfected with miRNA antisense sequence mimics were set as a negative control group (miR-SCR), and those transfected with miR-376c mimics were set as an experimental group (miR-376c). When the confluence reached 80%–90%, the cells were transfected with miR-SCR mimics or miR-376c mimics plasmids according to the manufacturer’s instructions for Lipofectamine^®^ 2000 reagent. The transfected cells were cultured for 4 h and then the medium was replaced with complete medium. Saos-2 cells with overexpressed miR-376c were eventually obtained after G418 screening and passage.

### Treatment of Saos-2 cells with cisplatin

Saos-2 cells were adjusted to the density of 2.5 × 10^5^/mL and treated with different doses of cisplatin (0, 2.5, 5.0, and 10.0 mg/L) for 48 h.

### Western blot

Saos-2 cells were digested, centrifuged, collected, and lysed with radioimmunoprecipitation assay (RIPA) buffer. The total protein concentration was determined according to the instructions of BCA kit (Beyotime Institute of Biotechnology, Shanghai, China). Each sample (50 mg) was subjected to SDS-PAGE, and the product was electrophoretically transferred onto a nitrocellulose membrane that was thereafter blocked with 5% skimmed milk at room temperature for 1 h and incubated with the corresponding primary antibody against TGF-α (1:1000 diluted) or β-actin [1:1000 diluted] (Abcam, USA) overnight at 4°C. The membrane was then washed and incubated with horseradish peroxidase (HRP)-labeled anti-mouse secondary antibody [1:5000 diluted] (Abcam, USA) at 37°C for 1 h. Scanning was conducted after enhanced chemiluminescence (ECL) development, and the relative protein expression was analyzed by Quantity-One software (Bio-Rad, USA) after internal reference calibration.

### Quantitative reverse transcription PCR (qRT-PCR)

When the confluence reached 80%–90%, Saos-2 cells were digested, centrifuged, and collected. RNA was then extracted and reverse transcribed into cDNA according to the instructions of M-MLV kit (Promega, USA). The target genes were amplified using cDNA and corresponding primers, to detect their expression in cells. The upstream primer for *TGFA* was 5’-GCCAACGTCAGTGAGGCAGA-3’ and that for miR-376c was 5’-ATAGAGGAAATTCCACGT-3’. The expression of *TGFA* and miR-376c was determined by a TaqMan detection kit (Thermo Fisher Scientific, USA). Real-time PCR was carried out using SYBR Green II fluorescent dye and IQ5TM real-time PCR system (Bio-Rad, USA), and the results were analyzed using U6 as the internal reference. The upstream primer for U6 was 5’-CGCAAGGATGACACGCAAATTC-3’. Relative expression was determined using the 2^-∆∆Ct^ method. The experiment was performed in triplicate.

### MTT assay

After incubation in a 6-well plate for 24 h, Saos-2 cells were digested, centrifuged, and adjusted to a final density of 1 × 10^5^/mL in a 96-well plate, with 100 μL per well. The MTT assay was performed on days 1–5, and the optical density at 490 nm was measured by a microplate reader (PerkinElmer, USA).

### BrdU assay

Saos-2 cells were seeded in a Petri dish with a diameter of 35 mm (with a coverslip inside) at 1.5 × 10^5^/mL, cultured for one day, and synchronized with medium containing 0.4% FBS for three days, leaving most cells in the G_0_ phase. Before the culture ended, BrdU (Thermo Fisher Scientific, USA; final concentration: 30 mg/L) was added for incubation at 37°C for 40 min. The medium was then discarded, and the coverslip was washed three times with PBS and fixed with methanol/acetic acid for 10 min. Endogenous oxidase was inactivated by 0.3% hydrogen peroxide-methanol for 30 min after air drying of the fixed coverslip. Subsequently, the coverslip was blocked with 5% normal rabbit serum (Abcam, USA), and nucleic acid was denatured with formamide at 100°C for 5 min. After being cooled on an ice-bath, the coverslip was washed with PBS, and anti-mouse BrdU monoclonal antibody (Abcam, USA; working concentration: 1:50) was added. Meanwhile, PBS or serum was added to negative control. The avidin–biotin complex (ABC) method was employed for detection. Hematoxylin and eosin (HE) staining was carried out. Total cells and BrdU-positive cells in 10 randomly selected high-magnification visual fields were counted under a light microscope (Olympus, Japan), and the labeling index was calculated.

### Luciferase reporter assay

The luciferase activity of samples was detected by E1910 Dual Luciferase Reporter Assay System (Promega, USA). The culture medium was discarded 48 h after transfection, and cells were washed twice with PBS. Afterwards, the cells in each well were added 100 μL of passive lysis buffer and gently shaken at room temperature for 15 min to collect the lysate. Afterwards, 20 μL of the lysate was added into GloMax microplate luminometer (Promega, USA). After measurement of the background for 2 s, 100 μL of LARII solution was added into each sample, quickly mixed and measured for 2 s. Subsequently, 100 μL of Stop and Glo reagent was added, rapidly mixed and measured for 2 s.

### Statistical analysis

All experiments were repeated three times independently. All data were analyzed using IBM SPSS Statistics for Windows, Version 20.0. (IBM Corp., Armonk, NY). Categorical data were expressed as mean ± standard deviation (SD). Comparisons between two groups were performed by the independent samples t-test, and those among multiple groups were conducted by one-way analysis of variance with completely random design. In the case of homogeneity of variance, the F test was performed, and the Bonferroni method was used for multiple comparison. In the case of heterogeneity of variance, the Welch approximate F test was conducted, and the Dunnett’s T3 method was utilized for multiple comparison. A value of *p <* 0.05 was considered statistically significant.

## RESULTS

### Correlation between miR-376c and TGFA expression in osteosarcoma tissues

**FIGURE 1:**
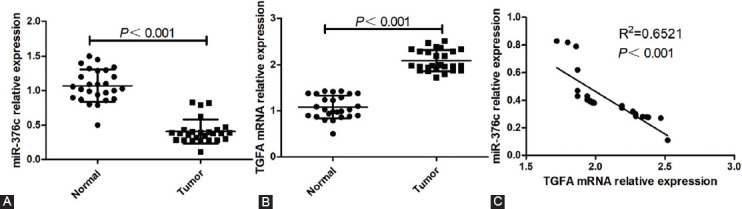
Correlation between miR-376c and *TGFA* expression in osteosarcoma tissues. (A) MiR-376c expression was significantly lower in osteosarcoma vs. normal skeletal muscle tissues; (B) *TGFA* expression was significantly higher in osteosarcoma vs. normal skeletal muscle tissues; (C) miR-376c and *TGFA* expression in osteosarcoma tissues showed a significant negative correlation. *TGFA*: Transforming growth factor alpha.

The relative expression of miR-376c in osteosarcoma tissue was significantly lower compared with normal skeletal muscle tissue (*p <* 0.001, Figure 1A), and the expression of *TGFA* mRNA in osteosarcoma tissue was significantly higher compared with normal tissue (*p <* 0.001, Figure 1B). MiR-376c and *TGFA* mRNA expression in osteosarcoma tissues showed a significant negative correlation (*p <* 0.001; Figure 1C).

### Expression of miR-376c and TGFA in Saos-2 and hFOB cells

**FIGURE 2:**
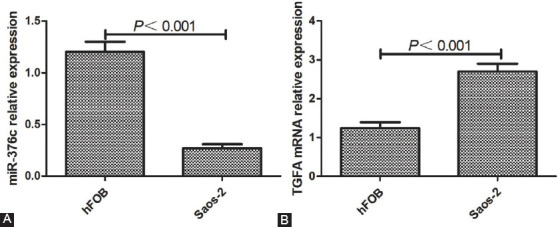
Expression of miR-376c and *TGFA* in Saos-2 and hFOB cells. (A) The expression of miR-376c in Saos-2 cells was significantly lower compared with hFOB cells, and (B) the expression of *TGFA* mRNA in Saos-2 cells was significantly higher compared with hFOB cells. *TGFA*: Transforming growth factor alpha.

The relative expression of miR-376c in Saos-2 cells was significantly lower compared with hFOB cells (*p <* 0.001, Figure 2A), and the relative expression of *TGFA* mRNA in Saos-2 cells was significantly higher compared with hFOB cells (*p <* 0.001; Figure 2B).

### Effects of TGFA on growth and proliferation of Saos-2 cells

**FIGURE 3:**
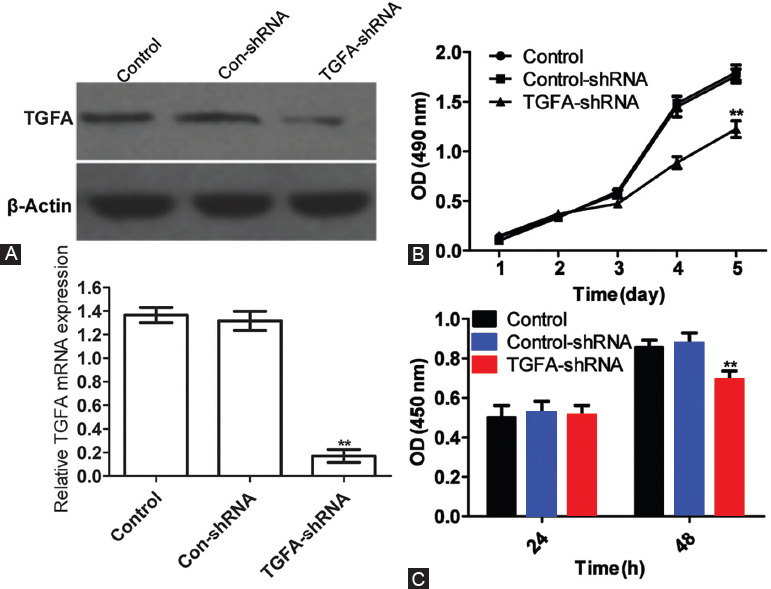
Effects of *TGFA* on growth and proliferation of Saos-2 cells. (A) Western blot and quantitative reverse transcription PCR (qRT-PCR) results of *TGFA* protein and mRNA expression, respectively, in Saos-2 cells with stably silenced *TGFA* expression. β-actin was used as the internal reference for Western blot and U6 for qRT-PCR. Relative expression in qRT-PCR was calculated according to the 2^-∆∆Ct^ method. The expression of TGF-α protein in *TGFA*-shRNA group was significantly lower compared with control-shRNA group. (B) Cell growth curves were plotted based on MTT assay results. Saos-2 cells were transfected with control-shRNA or *TGFA*-shRNA plasmids, transferred to 96-well plates 48 h later, stained by adding MTT every other day, and measured at 490 nm by microplate reader. The detection was performed for 5 consecutive days. The transfection with *TGFA*-shRNA significantly inhibited the growth of Saos-2 cells. (C) Cell proliferation was detected by BrdU assay. Saos-2 cells were transfected with control-shRNA or *TGFA*-shRNA plasmids and incubated with BrdU for 48 h. After 24 h, BrdU antibody was added into each group that was measured at 450 nm by microplate reader. The cells were detected again with the same method 48 h later. At 24 h, the proliferative capacities of cells were similar in all groups, but at 48 h, the proliferative capacity of *TGFA*-shRNA group was significantly decreased. Each sample was tested in triplicate and represented as mean ± SD. **Comparison between *TGFA*-shRNA and control-shRNA group, *p <* 0.05. *TGFA*: Transforming growth factor alpha; shRNA: Short hairpin RNA.

The qRT- PCR showed that *TGFA* mRNA expression in *TGFA*-shRNA group was downregulated by 80% compared with control-shRNA group, indicating that the transfection with *TGFA*-shRNA significantly inhibited the expression of *TGFA* (*p <* 0.05). The Western blot showed that the expression of TGF-α protein in *TGFA*-shRNA group was significantly lower compared with control-shRNA group (*p <* 0.05; Figure 3A). Therefore, Saos-2 cells with stably silenced *TGFA* expression were successfully constructed. As shown in Figure 3B, the transfection with *TGFA*-shRNA significantly inhibited the growth of Saos-2 cells (*p <* 0.05). At 24 h, the proliferative capacities of cells were similar in all groups, but at 48 h, the proliferative capacity of *TGFA*-shRNA group was significantly decreased (*p <* 0.05; Figure 3C). Thus, *TGFA* promoted the proliferation of Saos-2 cells.

### Effects of cisplatin on TGFA protein expression

**FIGURE 4:**
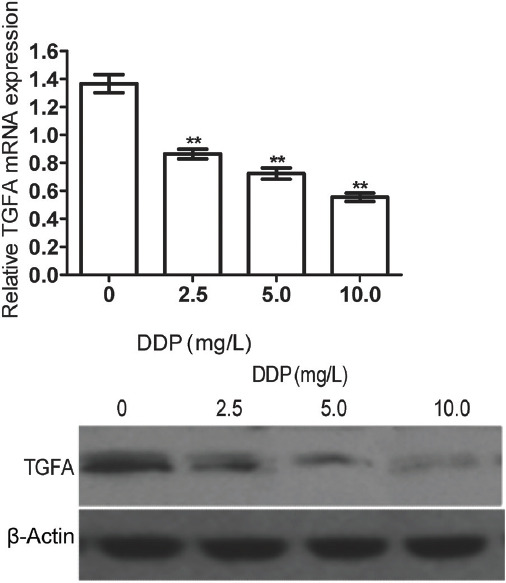
Quantitative reverse transcription PCR (qRT-PCR) and Western blot results of *TGFA* mRNA and protein expression, respectively, after cisplatin treatment. β-actin was used as the internal reference for Western blot and U6 for qRT-PCR. Relative expression in qRT-PCR was calculated according to the 2^-∆∆Ct^ method. Both *TGFA* mRNA and protein expression decreased with increasing cisplatin concentration. The experiments were performed in triplicate and represented as mean ± SD. **Compared with cells untreated with cisplatin, *p <* 0.05. DDP: Cisplatin; *TGFA*: Transforming growth factor alpha.

To evaluate the effects of cisplatin on *TGFA*, a pre-experiment was first carried out, finding that the EC_50_ value was 8 mg/L. We treated Saos-2 cells with 0, 2.5, 5.0, and 10.0 mg/L cisplatin for 48 h. The qRT- PCR and Western blot revealed that both *TGFA* mRNA and protein expression decreased with increasing cisplatin concentration (Figure 4). Accordingly, cisplatin significantly suppressed *TGFA* protein expression (*p <* 0.05).

### Cisplatin inhibited Saos-2 cell growth by downregulating TGFA expression

**FIGURE 5:**
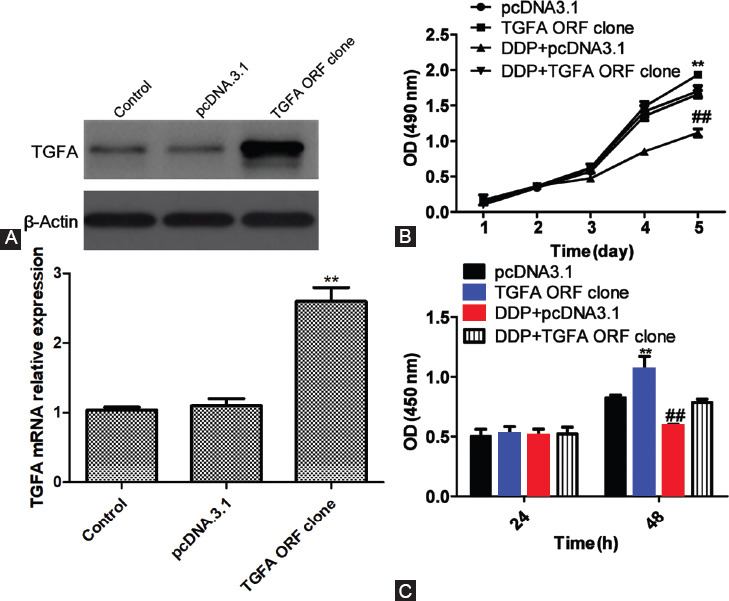
Effects of cisplatin and TGFA on Saos-2 cell growth and proliferation. (A) Western blot and quantitative reverse transcription PCR (qRT-PCR) results of TGFA protein and mRNA expression, respectively, in Saos-2 cells with stable TGFA overexpression. β-actin was used as the internal reference for Western blot and U6 for qRT-PCR. Relative expression in qRT-PCR was calculated according to the 2^-∆∆Ct^ method. Both TGFA mRNA and protein expressions in TGFA ORF clone group were significantly upregulated compared with pcDNA3.1 group. (B) Cell growth curves were plotted based on MTT assay results. Saos-2 cells were transfected with pcDNA3.1 and TGFA ORF clone, transferred to 96-well plates 48 h later, stained by adding MTT every other day, and measured at 490 nm by microplate reader. The detection was performed for 5 consecutive days. Compared with pcDNA3.1 group, the growth of cells in TGFA ORF clone group was significantly facilitated. After treatment with cisplatin (10 mg/L), the growth of cells in pcDNA3.1 group was significantly suppressed. (C) Cell proliferation was detected by BrdU assay. Saos-2 cells were transfected with pcDNA3.1 and TGFA ORF clone and incubated with BrdU for 48 h. After 24 h, BrdU antibody was added into each group that was measured at 450 nm by microplate reader. The cells were detected again with the same method 48 h later. The proliferative capacities of all groups were similar at 24 h. Each sample was tested in triplicate and represented as mean ± SD. **Comparison between TGFA ORF clone and pcDNA3.1 groups, *p <* 0.05; ^##^comparison between cisplatin + pcDNA3.1 and pcDNA3.1 groups, *p <* 0.05. DDP: Cisplatin; TGFA: Transforming growth factor alpha; shRNA: Short hairpin RNA; ORF: Open reading frame.

The qRT- PCR and Western blot showed that both *TGFA* mRNA and protein expressions in *TGFA* ORF clone group were significantly upregulated compared with pcDNA3.1 group (*p <* 0.05; Figure 5A). Hence, Saos-2 cells with stable overexpression of *TGFA* were successfully constructed [9].

Compared with pcDNA3.1 group, the growth of cells in *TGFA* ORF clone group was significantly facilitated (*p <* 0.05). After treatment with cisplatin (10 mg/L), the growth of cells in pcDNA3.1 group was significantly suppressed (*p <* 0.05; Figure 5B). The proliferative capacities of all groups were similar at 24 h (Figure 5C). Collectively, cisplatin inhibited Saos-2 cell proliferation by downregulating *TGFA* expression.

### Effects of cisplatin on miR-376c expression

**FIGURE 6:**
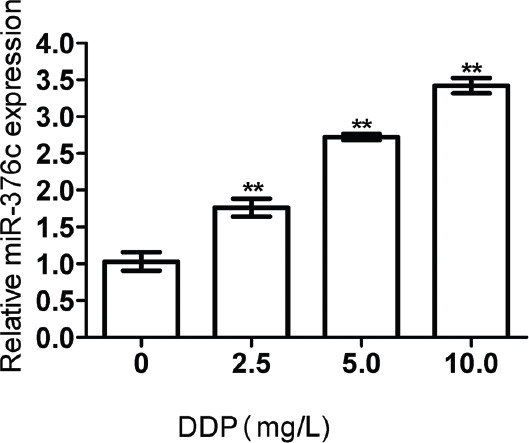
MiR-376c expression after cisplatin treatment detected by quantitative reverse transcription PCR. U6 was used as the internal reference. Relative expression was calculated according to the 2^-∆∆Ct^ method. With increasing cisplatin concentration, miR-376c expression significantly increased after 48 h of treatment. The experiments were performed in triplicate and represented as mean ± SD. **Compared with cells untreated with cisplatin, *p <* 0.05. DDP: Cisplatin.

With increasing cisplatin concentration, miR-376c expression significantly increased after 48 h of treatment (*p <* 0.05; Figure 6), suggesting that cisplatin promoted miR-376c expression.

### Cisplatin inhibited Saos-2 cell growth by upregulating miR-376c expression

**FIGURE 7:**
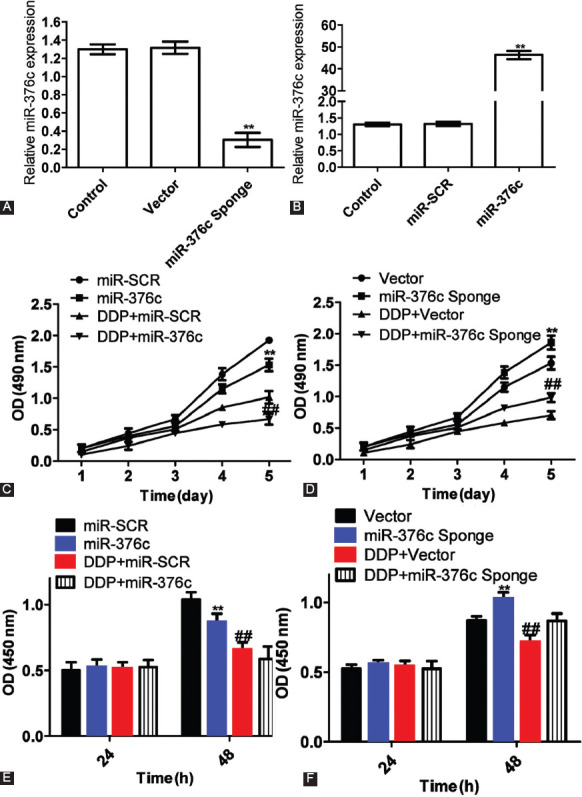
Cisplatin inhibited Saos-2 cell growth by upregulating miR-376c expression. (A) MiR-376c expression after transfection with miR-376c sponge detected by quantitative reverse transcription PCR (qRT-PCR). MiR-376c expression in miR-376c sponge group was downregulated by approximately 60% compared with vector group. **Compared with vector group, p < 0.05. (B) MiR-376c expression after transfection with miR-376c mimics detected by qRT-PCR. MiR-376c mimics increased miR-376c expression about 47-fold compared with miR-SCR group (negative control). **Compared with miR-SCR group, p < 0.05. U6 was used as the internal reference for qRT- PCR. Relative expression was calculated according to the 2^-∆∆Ct^ method. (C) Effects of cisplatin and miR-376c on Saos-2 cell growth. The growth of Saos-2 cells was suppressed most significantly by combining cisplatin (10 mg/L) treatment with miR-376c transfection. **Comparison between miR-376c and miR-SCR group, p < 0.05; ^##^comparison between cisplatin + miR-376c and cisplatin + miR-SCR group, p < 0.05. (D) Effects of cisplatin and miR-376c sponge on Saos-2 cell growth. The transfection with miR-376c sponge facilitated cell growth. The cell growth was suppressed by cisplatin plus control vector, which was reversed by combining cisplatin with miR-376c sponge. **Comparison between miR-376c sponge and vector group, p < 0.05; ^##^comparison between cisplatin + miR-376c sponge and cisplatin + vector group, p < 0.05. (E) Effects of cisplatin and miR-376c on Saos-2 cell proliferation. Cisplatin inhibited cell proliferation. **Comparison between miR-376c and miR-SCR group, p < 0.05; ^##^comparison between cisplatin + miR-376c and cisplatin + miR-SCR group. (F) Effects of cisplatin and miR-376c sponge on Saos-2 cell proliferation. Cisplatin inhibited cell proliferation. **Comparison between miR-376c sponge and vector groups, p < 0.05; ^##^ comparison between cisplatin + miR-376c sponge and cisplatin + vector group, p < 0.05. The experiments were performed in triplicate and represented as mean ± SD. DDP: Cisplatin.

MiR-376c expression in miR-376c sponge group was downregulated by approximately 60% compared with vector group (Figure 7A), thus, the transfection with miR-376c sponge significantly suppressed miR-376c expression (*p <* 0.05). MiR-376c mimics increased miR-376c expression about 47-fold compared with miR-SCR group (Figure 7B). Therefore, the transfection with miR-376c mimics significantly upregulated the expression of miR-376c (*p <* 0.05).

The MTT assay showed that the growth of Saos-2 cells was suppressed most significantly by combining cisplatin (10 mg/L) treatment with miR-376c transfection (*p <* 0.05; Figure 7C). As displayed in Figure 7D, the transfection with miR-376c sponge facilitated cell growth, further verifying the growth-inhibitory effects of miR-376c. The cell growth was suppressed by cisplatin plus control vector, which was reversed by combining cisplatin with miR-376c sponge. Thus, cisplatin suppressed cell growth by upregulating miR-376c expression.

The proliferative capacities of Saos-2 cells followed a similar trend (Figure 7E and F), suggesting that cisplatin inhibited cell proliferation by upregulating miR-376c expression.

### Targeted regulatory effects of miR-376c on TGFA

### Cisplatin inhibited Saos-2 cell growth by downregulating TGFA expression

The qRT- PCR and Western blot showed that both *TGFA* mRNA and protein expressions in *TGFA* ORF clone group were significantly upregulated compared with pcDNA3.1 group (*p <* 0.05; Figure 5A). Hence, Saos-2 cells with stable overexpression of *TGFA* were successfully constructed [9].

Compared with pcDNA3.1 group, the growth of cells in *TGFA* ORF clone group was significantly facilitated (*p <* 0.05). After treatment with cisplatin (10 mg/L), the growth of cells in pcDNA3.1 group was significantly suppressed (*p <* 0.05; Figure 5B). The proliferative capacities of all groups were similar at 24 h (Figure 5C). Collectively, cisplatin inhibited Saos-2 cell proliferation by downregulating *TGFA* expression.

### Effects of cisplatin on miR-376c expression

With increasing cisplatin concentration, miR-376c expression significantly increased after 48 h of treatment (*p <* 0.05; Figure 6), suggesting that cisplatin promoted miR-376c expression.

### Cisplatin inhibited Saos-2 cell growth by upregulating miR-376c expression

MiR-376c expression in miR-376c sponge group was downregulated by approximately 60% compared with vector group (Figure 7A), thus, the transfection with miR-376c sponge significantly suppressed miR-376c expression (*p <* 0.05). MiR-376c mimics increased miR-376c expression about 47-fold compared with miR-SCR group (Figure 7B). Therefore, the transfection with miR-376c mimics significantly upregulated the expression of miR-376c (*p <* 0.05).

The MTT assay showed that the growth of Saos-2 cells was suppressed most significantly by combining cisplatin (10 mg/L) treatment with miR-376c transfection (*p <* 0.05; Figure 7C). As displayed in Figure 7D, the transfection with miR-376c sponge facilitated cell growth, further verifying the growth-inhibitory effects of miR-376c. The cell growth was suppressed by cisplatin plus control vector, which was reversed by combining cisplatin with miR-376c sponge. Thus, cisplatin suppressed cell growth by upregulating miR-376c expression.

The proliferative capacities of Saos-2 cells followed a similar trend (Figure 7E and F), suggesting that cisplatin inhibited cell proliferation by upregulating miR-376c expression.

### Targeted regulatory effects of miR-376c on TGFA

**FIGURE 8:**
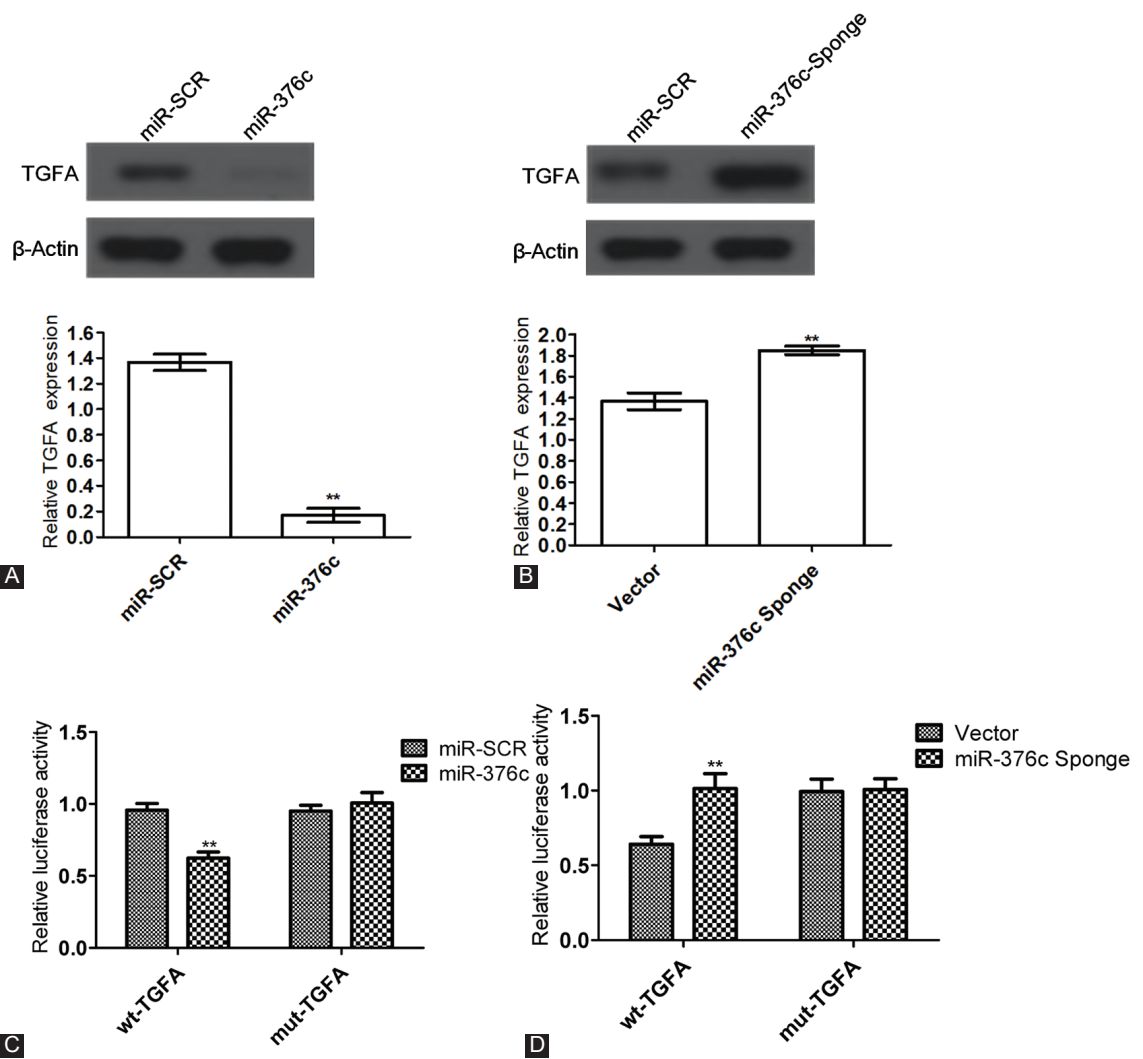
Targeted regulatory effects of miR-376c on *TGFA*. (A) Effects of overexpression of miR-376c on *TGFA* protein expression. *TGFA* protein expression in miR-376c group was significantly downregulated compared with miR-SCR group. (B) Effects of downregulation of miR-376c on *TGFA* protein expression. *TGFA* protein expression in miR-376c sponge group was significantly upregulated compared with vector group. (C and D) Dual luciferase reporter assay results. The luciferase activity of wild-type *TGFA* 3’ UTR reporter plasmid was only 70% of that in control group, but the activity in miR-376c sponge group was 1.3-fold that of control group. In contrast, the luciferase activities of mutant *TGFA* 3’ UTR reporter plasmid in the two transfection groups were similar to that in control group. The experiments were performed in triplicate and represented as mean ± SD. **p < 0.05. *TGFA*: Transforming growth factor alpha; UTR: Untranslated region.

*TGFA* protein expression in miR-376c group was significantly downregulated compared with miR-SCR group (*p <* 0.05; Figure 8A), and *TGFA* protein expression in miR-376c sponge group was significantly upregulated compared with vector group (*p <* 0.05; Figure 8B). Accordingly, miR-376c effectively inhibited *TGFA* protein expression, and the expressions of miR-376c and *TGFA* were negatively associated. The results are in agreement with those of Jin et al. [9].

The 1000-bp 3’ untranslated region (UTR) region of *TGFA* is near the binding site of wild-type miR-376c. Meanwhile, the binding site that was predicted by online tools such as TargetScan and miRanda was mutated (i.e. from UCUAUGU to UAUCUAU in the 3’-end sequence). The luciferase activity of wild-type *TGFA* 3’ UTR reporter plasmid was only 70% of that in control group (Figure 8C and D), but the activity in miR-376c sponge group was 1.3-fold that of control group. In contrast, the luciferase activities of mutant *TGFA* 3’ UTR reporter plasmid in the two transfection groups were similar to that in control group. Taken together, miR-376c inhibited *TGFA* expression by directly interacting with its 3’ UTR region. Similar to the results of Jin et al. [9], we showed that miR-376c can suppress *TGFA* expression in a targeted way.

## DISCUSSION

*TGFA* is highly expressed in cancers of the digestive tract, the reproductive system, and the lungs, with the levels recovered to normal after tumor resection [14]. TGF-α can interact with several growth factors and receptors to result in phenotype transformation, so it has become a target for the diagnosis and treatment of different types of cancers [9]. Previously, it has been found that *TGFA* has a critical role in the progression of osteosarcoma [15].

The expression of a specific gene in mammals can be inhibited by small interfering RNA (siRNA) through direct RNA interference. However, it is still challenging to prepare a specific siRNA for targeted gene silencing [16]. In this study, the inhibition of *TGFA* expression suppressed Saos-2 cell growth and DNA synthesis, which was reversed by cisplatin treatment. Therefore, *TGFA* played a crucial role in Saos-2 cell proliferation. Our results provide valuable evidence for augmenting the sensitivity of osteosarcoma to chemotherapeutic agents.

Cisplatin affects tumor cell proliferation and growth depending on the type and origin of cells as well as the dose and time of the treatment [17]. Fellenberg et al. found that the growth of liver cancer cells was obviously inhibited with increasing dose of cisplatin. Low- and high-dose cisplatin treatments arrested the cells in the G0-G1 phase and the S-G2 phase, respectively [18]. Therefore, cisplatin affects cell proliferation in different ways. In this study, after cisplatin treatment, the growth of Saos-2 cells was significantly suppressed and miR-376c expression was increased. However, these effects were reversed by transfection with miR-376c sponge targeting miR-376c. In other words, Saos-2 cells stopped growing due to miR-376c expression, thereby becoming sensitive to the chemotherapeutic agent.

In animals, miRNA commonly undergoes incomplete base pairing with the 3’ UTR region of its target gene [19]. It is rather difficult to identify the binding site of miRNA, which requires experimental methods (e.g. RT-PCR, Western blot, and luciferase reporter assay) performed based on theoretical prediction of target gene using bioinformatics tools. In this study, we conducted the dual luciferase reporter assay [20] to determine whether miR-376c exerts targeted inhibitory effects on *TGFA* in Saos-2 cells. *TGFA* was predicted to be a potential target gene of miR-376c, with a binding site in the 3’ UTR region [21]. Accordingly, *TGFA* expression was downregulated probably because of upregulation of miR-376c expression. Meanwhile, it is necessary to clarify the role of miR-376c in the proliferation, differentiation, and apoptosis of osteosarcoma cells, as well as the mechanisms underlying the onset and progression of this cancer. The luciferase activity was inhibited by overexpressing miR-376c and was enhanced by silencing miR-376c, but neither of the two processes occurred after the binding site of miR-376c was mutated. Hence, miR-376c indeed regulated *TGFA* in a targeted way.

## CONCLUSION

In summary, cisplatin inhibited the proliferation of osteosarcoma cells Saos-2 by downregulating *TGFA* and upregulating miR-376c. Given that miR-376c exerted targeted inhibitory effects on *TGFA* expression, cisplatin may suppress the proliferation of osteosarcoma cells via the miR-376c/*TGFA* pathway.
